# Vaccination Upregulates Th1 Cytokines in the Lung of Pigs Experimentally Infected with *Mycoplasma hyopneumoniae*

**DOI:** 10.3390/ani13030520

**Published:** 2023-02-01

**Authors:** Francisco Rodríguez, Rubén S. Rosales, Ana S. Ramírez, José B. Poveda

**Affiliations:** Instituto Universitario de Sanidad Animal y Seguridad Alimentaria, Veterinary Faculty, University of Las Palmas de Gran Canaria, Trasmontaña S/N, 35413 Arucas, Spain

**Keywords:** cytokines, immunohistochemistry, *Mycoplasma hyopneumoniae*, porcine, pneumonia, vaccination

## Abstract

**Simple Summary:**

*Mycoplasma hyopneumoniae* (Mhy) is the cause of porcine enzootic pneumonia (PEN) which, although not lethal, may lead to fatal pneumonia caused by opportunistic bacteria and/or viruses, in concurrence with environmental and management stressors due to reduced muco-ciliary clearance and impaired immune-inflammatory function. PEN spreads progressively with high morbidity rate, causing major economic losses to pig producers worldwide, associated to diminished production indexes and increased control costs, based on vaccination and antimicrobial treatments, used to mitigate those detrimental effects. Vaccination is commonly used for Mhy prophylaxis. While vaccines improve growth performance and reduce clinical signs and lung lesions, the results are often variable. In response to multiple stimuli, cytokines -molecular signals produced by a variety of immuno-modulatory cells- are involved in numerous protective biological activities. To investigate the role of cytokines in vaccinated and non-vaccinated pigs, an experimental model of infection was developed, by combining trans-tracheal and aerosol route, using a mixture of Mhy-field strains. Comparison of clinical, microbiological, pathological, and immunohistochemical parameters revealed significant differences depending upon the profile of cytokines expressed. These results indicate the importance to enhance Th1-type cytokine response—cell-mediated immunity -, to improve the effectiveness of Mhy-immunization, and to develop more efficient vaccines against PEN.

**Abstract:**

*Mycoplasma hyopneumoniae* (Mhy) is the causative agent of enzootic pneumonia, characterized by high morbidity and low mortality rates in intensive swine production systems. To better understand the mechanisms underlying the protection of an inactivated whole cell vaccine, we investigated the immunohistochemical differences in the cytokine expression in vaccinated and non-vaccinated pigs experimentally infected with Mhy. Four-week-old Mhy-negative pigs (*n* = 24) were allocated to negative control (*n* = 8), or one of two Mhy-infected groups: vaccinated (*n* = 8) and non-vaccinated (*n* = 8). Infection was carried out by a combination of trans-tracheal and aerosol route. Lung samples were processed for histopathological and immunohistochemical studies, by using antibodies against Mhy, IL1-α, IL1-β, IL-2, IL-4, IL-5, IL-6, Il-8, IL-10, IL-12p35, IL-13, IL-17A, TNF-α, IFN-γ, and CD-4 lymphocytes. Although all cytokines increased in both infected groups, IL-2, IL-12, and IFN-γ were significantly overexpressed in vaccinated pigs. These findings, in conjunction with the decrease of macroscopic and histological lesions in vaccinated animals, indicate the importance to enhance Th1 response in the immunization strategies to control Mhy infection.

## 1. Introduction

*Mycoplasma hyopneumoniae* (Mhy) is the etiologic agent of porcine enzootic pneumonia (PEN) [1]. The disease, characterized by chronic non-productive coughing and poor growth rate, occurs worldwide and causes significant economic losses [2]. Colonization of the lower respiratory tract by Mhy frequently results in lung lesions characterized by variably sized areas of dark consolidation in the apical, cardiac, and intermediate lobes [3]. Histological changes in the acute phase of Mhy infection involve loss of cilia from the respiratory epithelial cells, exfoliation, and buildup of neutrophils and macrophages inside and in the periphery of the airways [4,5]. The changes associated to the evolution of the disease consist of peribronchial or peribronchiolar lymphocytes and macrophages infiltrates, exudation of inflammatory cells in the airways, and hyperplasia of the lymphoid tissue associated with intrapulmonary airways, known as bronchus-associated lymphoid tissue (BALT) [5,6].

On-farm control and eradication plans are frequently carried out to combat Mhy infection in porcine production worldwide. Improved management strategies, vaccine-based prophylaxis, and antibiotic therapy is used, alone or in combination, to reduce the detrimental outcomes of Mhy infection [7]. Some of the advantages described in connection with Mhy vaccination comprise improvement of production indicators, reduction in the time to obtain slaughter weight, decrease in clinical signs and lung lesions, lower mortality, improvement of carcass quality, and reduction of microorganisms in the respiratory tract [8]. Vaccines are used worldwide and mainly consist of intramuscularly administered inactivated whole-cell preparations. Although vaccination confers overall beneficial effects, the results are variable depending, among others, of infection levels, circulating strains, and unknown aspects related with the induced immune responses [2,7,9].

Mhy induces the production of several cytokines by macrophages and other immune or non-immune cells, detected in bronchoalveolar lavage fluid and lung tissue of infected pigs, which have been implicated in the pathogenesis of PEN, and in the development of lesions in the lung and respiratory tract of infected animals [10,11,12]. Inflammatory cytokines activate the immune system in response to danger and increase the efficiency of an immune response by enhancing antigen presenting cell function [13]. Several cytokines, such as Interleukin (IL)-1α, IL-1β, IL-2, IL-4, IL-6, IL-8, IL-10, IL-12, Interferon (INF)-γ, and Tumor Necrosis Factor (TNF)-α are shown to be produced at pneumonic sites by immunohistochemistry [14,15]. TNF-α, IL-1, and IL-6 have been shown to be associated with the development of Mhy-pneumonia under experimentally conditions [9,10], being these cytokines involved in the prominent lymphoplasmacytic infiltration in pneumonic areas [16]. Finally, overexpression of these cytokines in pulmonary alveolar macrophages and bronchial lavage fluid of pigs infected with both Mhy and porcine reproductive and respiratory syndrome virus, appeared to be related with the capacity of the respiratory tract to eliminate these pathogens during PEN [17]. Abilities to modulate and/or to elude the host’s immune response by Mhy have been described as vital strategies that enable this microorganism to endure and, subsequently, to produce sustained inflammation, resulting in relevant lung lesions [18,19].

Our study intended to establish the potential association between vaccination, clinical and pathological findings, and cytokine expression in the lungs of pigs experimentally infected with Mhy.

## 2. Materials and Methods

### 2.1. Animals, Housing and Experimental Design

Twenty-four Large White pigs of 30 days of age, males and females, and originating from a certified Mhy-free farm were utilized as part of this study. Animals were identified with ear tags and randomly allocated in 3 groups (C0, C1 and C2) with 8 piglets each. Each group was kept in separated identical boxes, to prevent the risk of pathogen spread between groups during the experiment, provided with 16 m^2^ area with filtered air. Animals were kept in a 15 h light/9 h dark regimen, they had unlimited access to water, and were fed with feed from a local supplier. Nasal swabs were taken on arrival from all piglets to demonstrate the absence of Mhy in the upper respiratory tract by qPCR, following the protocol described by Marois et al. [20] using a BioRad CFX96 thermal cycler. In addition, prophylaxis with Tulathromycin (DRAXXIN^®^, Louvain-la-Neuve, Belgium, 0.025 mL/Kg, intramuscular (IM)) was performed on arrival.

### 2.2. Experimental Inoculation

Piglets in group C1 were vaccinated, by the intramuscular route in the neck, with 2 mL of Mhy chemically-inactivated saponin-adjuvated whole bacteria vaccine containing two local strains. Piglets in group C2 were non-vaccinated and kept as infection controls. Twenty-three days post-vaccination pigs of groups C1 and C2 were inoculated, by transtracheal route using 10 mL of a pool of cultures including seven Mhy field strains, filtered through a 0.22 μm pore size membrane. All strains were grown in modified Friis liquid medium and collected for inoculation in the logarithmic phase of growth. In addition, a Mhy aerosol was administered using an electric portable aerosol applicator (DYNA-FOG^®^ Hurricane Ultra) with a 20 μm diameter droplet, during 30 min in 3 consecutive days. Control group (C0) received 10 mL of sterile modified Friis liquid medium by transtracheal route. All Mhy cultures used for inoculation of the piglets were applied at a concentration of 10^5^ colour changing units (CCU).

Pigs were examined daily for 15 min to detect the appearance of respiratory signs, including cough, nasal discharge, and respiratory distress. Body condition or changes in normal behaviour were also examined. Cumulative daily clinical signs scoring can be found on Table 1.

### 2.3. Necropsy Procedures and Sample Collection

All pigs were euthanized 29 days after infection, by means intramuscular deep sedation with Zoletil™ (Virbac, Carros, France, 6 mg/kg), followed by intracardiac injection of pentobarbital (EUTHASOL^®^ 100 mg/kg, Dechra, Shrewsbury, UK). Lungs were taken and examined in order to determine the extension of gross lesions, and the results expressed, as previously described [21], as percentages of pulmonary consolidation. Microbiological, histological, and immunohistochemical analysis were carried out in samples from the cranial lung lobes of all animals studied.

For histological and immunohistochemical analysis, lung samples were fixed in neutral buffered formalin, embedded in paraffin, and serial 4 µm thickness slides obtained. Classification of histologic lesions was carried out in samples routinely stained with hematoxylin and eosin (HE), following the semiquantitative criteria described by Livingston et al. [4]. Lung lesion scores ranged from 0 to 4, where 0: negative; 1: indicates one or more lymphoid nodules involving the muscularis mucosa of bronchi and bronchioles; 2: indicates lymphoid nodules affecting the muscularis mucosa of bronchi and bronchioles as well as the presence of inflammatory cells in the septal wall, bronchus, and alveolar lumen; 3: indicates perivascular and peribronchiolar hyperplasia of the lymphoid tissue; and 4: indicates marked perivascular and peribronchiolar lymphoid hyperplasia in extensive areas of the lung parenchyma, with distortion of airways. In addition, density of alveolar macrophages was evaluated, based on the number of cells per mm^2^, as follows: 0: <20; 1: 20–40; 2: 40–60; and 3: >60.

### 2.4. Microbiological Analysis

Lung tissue samples were sampled using sterile viscose swabs. After that, the tip of the swabs was placed in Friis modified liquid broth and incubated for 24 h at 37 °C in constant stirring. Following the initial incubation, cultures were filtered using 0.45 μm membranes (Millipore, Pennsylvania, PA, USA) and incubated until a color change evidencing a pH shift was observed. Subcultures were performed on solid Friis medium, incubated at 37 °C in a humid chamber. Species-specific PCR [22] was used to detect the presence of Mhy and other relevant porcine mycoplasmas (*M. hyorhinis*, *M. hyosynoviae,* and *M. flocculare*) in culture samples. In addition, lung samples were also processed for general bacteriology using standard methods. Prior to culturing, lung tissue samples were processed for direct DNA extraction using E.Z.N.A.^®^ MicroElute^®^ Genomic DNA extraction Kit (Omega Bio-Tek, Norcross, GA, USA) following the manufacturer’s instructions, and the nucleic acid obtained used for Mhy bacterial load quantification by qPCR [20]. Statistical differences in bacterial load between experimental groups was assessed by independent sample *t*-test with SPSS Inc. 26.0 for Windows (IBM, Chicago, IL, USA). Differences were considered significant when *p* < 0.05.

### 2.5. Immunohistochemistry

For immunohistochemistry, sections were dewaxed and rehydrated, and endogenous peroxidase activity was blocked by incubation with 3% H_2_O_2_ in methanol for 30 min at room temperature. Tissues were subjected to heat induced antigen retrieval (water bath at 98 °C) with antigen retrieval solution pH 6.0 (Dako, Glostrup, Denmark) for 15 min. All sections were incubated with 10% rabbit or mouse normal serum (Vector Laboratories, Burlingame, CA, USA) for 30 min at room temperature. The primary monoclonal or polyclonal reagents, diluted 1:150, which included rabbit polyclonal anti-Mhy [5], and mouse monoclonal anti-IL-1α (clone C1), IL-1β (clone OTI3E1), IL-2 (clone C5), IL-4 (clone MP4-25D2), IL-5 (clone TRFK5), IL-6 (clone MP5 20F3), IL-8 (clone NAPII), IL-10 (clone JES3-12G8), IL-12p35 (clone C3), IL-13 (clone JES10-5A2), IL-17A (clone 41809),TNF-α (clone VPM61), IFN-γ (clone XMG1.2), and CD-4 (clone 4SM95) (Thermo Fisher Scientific, Inc., Waltham, MA, USA) antibodies, were then applied overnight at 4 °C. A biotinylated swine anti-rabbit or rabbit anti-mouse IgG (Vector Laboratories), diluted 1:200, was applied as secondary reagents for 30 min at room temperature. An ABC complex (Vector Laboratories) diluted 1:50 was applied as the third reagent. The sections were incubated for 3 min with 3,3’-diaminobenzidine tetrahydrochloride (Sigma, St. Louis, MO, USA) 0.035% in TBS containing H_2_O_2_ 0.1%. After rinsing in tap water, slides were lightly counterstained with Harris’s hematoxylin and mounted under DPX Mountant (BDH Laboratory Supplies, Poole, UK) for microscopy. Substitution of normal sera for the primary antibodies served as negative controls. Control tissues were processed alongside the test slides. Positive cells were counted in 40 non-overlapping and randomly selected high magnification fields of 0.237 mm^2^ from each sample and antibody. Results were expressed as means ± SD of positive cells per mm^2^, and analysis of variance for mean comparisons assessed by a nonparametric one-way ANOVA on ranks (Kruskal-Wallis) with Dunn´s post-hoc test for multiple pairwise comparisons between groups, with the SPSS Inc. 26.0 for Windows (IBM, Chicago, IL, USA). For parametric data, different groups were compared using two-tailed ANOVA and multiple comparisons were performed using Tukey test. Differences were considered significant when *p* < 0.05. The evaluation of the histological lesions, density of alveolar macrophages, and counting of cells positive for the different antibodies was carried out by one observed (FR), who was blinded to the group of animals to which each histological slide belonged.

## 3. Results

### 3.1. Physical Examination, Microbiological Analysis and Mhy Quantification

Clinical signs of non-productive spontaneous and frequent dry cough, characteristic of Mhy infection, was consistently present from 9 dpi until the end of the experiment in all animals of group C2 (Table 1). In addition, mild respiratory distress, characterized by a slightly increased resting respiratory rate, was also observed in those animals. Limited clinical respiratory signs were observed in group C1 (animals 9, 10 and 14). Uninfected (C0) animals did not present any clinical sign. No changes in body temperature were observed in any of the groups.

General bacteriological examination of the lung samples post-mortem did not demonstrate the presence of any common microorganism in the tissue samples analyzed. Likewise, molecular identification of *M. hyorhinis*, *M. hyosynoviae*, and *M. flocculare* was negative in all samples tested. Mhy was detected in lung tissue samples from all animals included in group C2. Mhy specific DNA was also detected in lung tissue samples from animals 12, 13, 14, and 15 belonging to group C1. All animals in group C0 were negative for the presence of Mhy DNA in lung tissue samples. Mhy detection was confirmed with both conventional [22] and specific qPCR [20]. Mhy PCR results, qPCR cycle threshold (Ct) and relative Mhy copy numbers can be found in Table 2. Relative Mhy copy number observed in group C2 (mean relative copy number = 1.01 × 10^5^) was significantly higher than group C1 (mean relative copy number = 1.09 × 10^2^) (*p* = 0.038).

### 3.2. Gross and Microscopic Lesions

All pigs of group C2 showed variable but typical well demarcated areas of dark red to purple, consolidated areas in the cranial, cardiac, and intermediate lobes of the lungs. The percentage of affected areas is presented in Table 1. Affected areas showed a catarrhal bronchointerstitial pneumonia, with development of peribronchial, peribronchiolar, and perivascular accumulations of lymphoid cells and formation of lymphoid follicles. In severe cases, the lymphoid nodules caused narrowing of the lumina of airways. Hyperplasia of goblet cells in bronchi and large bronchioles was commonly noticed. The thickening of alveolar septa was observed due to accumulation of lymphocytes, plasma cells, and neutrophils, and hyperplasia and hypertrophy of type II pneumocytes. Most animals showed a grade 4 of BALT hyperplasia (5 animals), and 2 pigs showed grade 2. In group C1, grade 1 of BALT hyperplasia was noted in 2 pigs (n° 12 and 13). An increase of alveolar macrophages (>40 cells/mm^2^) was observed in all pigs of group C2. Two pigs of group C0 (nos. 3 and 6) and four of group C1 (nos. 11, 12, 13, and 15) showed between 20–40 cells/mm^2^. Significant differences (*p* < 0.05) between vaccinated and un-vaccinated groups were detected in the scores for gross and microscopic lesions.

### 3.3. Immunohistochemistry

#### 3.3.1. Mhy Antigen Expression

**Figure 1:**
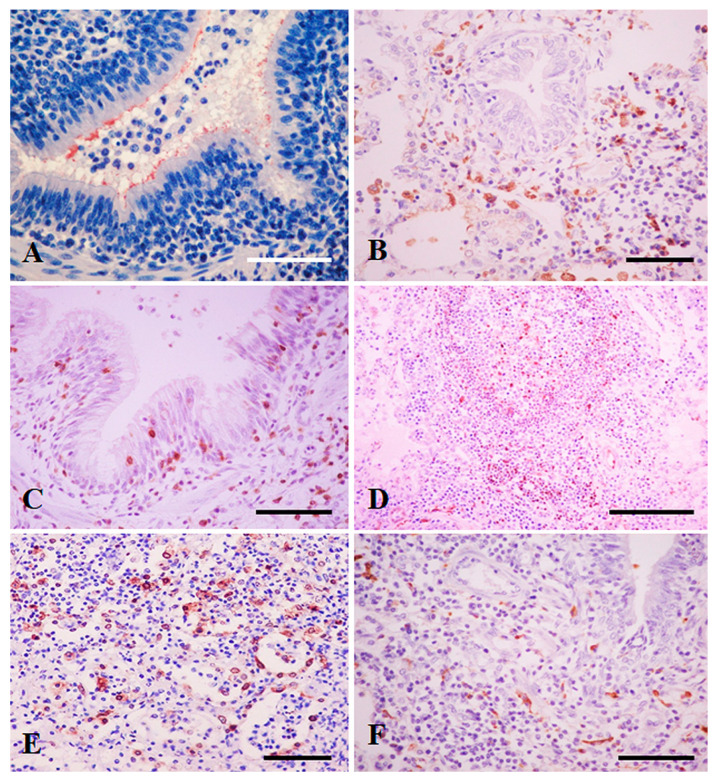
(**A**) Immunolabelling of Mhy-antigen on the surface of bronchial epithelial cells, pig n° 19. (**B**) Infiltration of macrophages and lymphocytes in alveolar spaces labelled for IL-12, pig n° 12. (**C**) Intraepithelial and peribronchiolar lymphocytes immunostained with IL-10 antibody, pig n° 11. (**D**) IL-2 reaction in lymphocytes of the BALT, pig n° 15. (**E**) IL-8 immunoreaction in alveolar exudate and pneumocytes, pig n° 23. (**F**) Cells with dendritic processes immunolabelled with INF-γ antibody, pig n° 14. Bars = 150 µm.

Numerous Mhy cells were immunolabeled, as a granular reaction, at the luminal surface of bronchial and bronchiolar epithelial cells of all pigs of group C2 (Figure 1A), and, with low intensity, in one pig (n° 12) of group C1. Mhy antigen was also seen in extracellular spaces combined with the mucus exudate, and within neutrophils and macrophages present in bronchial and bronchiolar lumina, and alveolar spaces. The remaining 15 pigs of group C0 and C1 resulted negative with this antibody.

#### 3.3.2. Cytokine Expression

**Figure 2:**
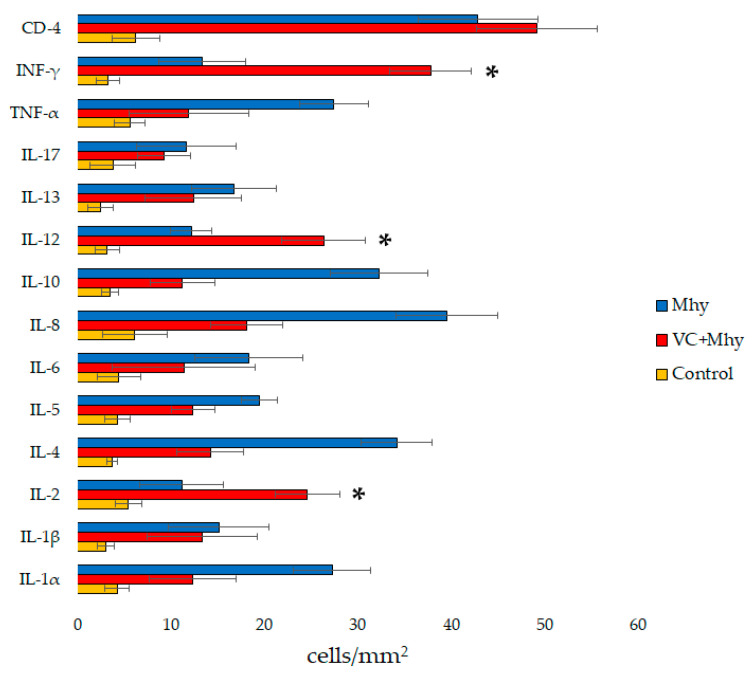
Immunohistochemical results, expressed as mean ± standard deviation of positive cells per mm^2^, in Control (un-vaccinated/uninfected), VC + Mhy (vaccinated/infected), and Mhy (un-vaccinated/infected) pigs. Significant (*) overexpressed Th1-type cytokines (IL-2, IL-12 and INF-γ) in vaccinated/infected pigs in relation with both un-vaccinated/uninfected and un-vaccinated/infected groups.

Figure 2 summarises the results obtained for the levels of cytokines analysed. The immunolabelling of cytokines was observed in areas of lung with bronchointerstitial pneumonia and in alveolar macrophages and was minimal in areas without lesions or macrophage infiltration. Immunoreactivity was detected in the cytoplasm of mononuclear cells within bronchiolar and alveolar lumina; randomly scattered in the alveolar septa; in the BALT, especially in lymphocytes of the perifollicular areas, and cells with long cytoplasmic processes (dendritic cells) of the germinal centres (Figure 1B–F). Immunoreactive cells generally showed large oval nuclei and abundant cytoplasm, or round nuclei with a narrow rim of cytoplasm. Scattered endothelial cells of the alveolar septa and neutrophils in bronchoalveolar spaces were also immunolabeled with IL-1α, IL-8, IL-17, and TNF-α antibodies.

## 4. Discussion

Mhy is considered one of the main pathogens in the porcine respiratory disease complex, by producing major economic losses associated to diminished production indexes and increased treatment and control costs, based on antimicrobial and vaccination treatments [1,23]. One of the main characteristics of PEN is the persistence of the mycoplasma infection and development of a chronic inflammatory response. The immune mechanisms involved in mycoplasma disease and resistance from infection still need much investigation in order to be fully understood. Research in mycoplasma respiratory diseases has shown that immune response is a dynamic process and involves a network of cellular and cytokine signals that determine the type of responses generated, and ultimately, the outcome of infection [2,24]. The current work developed an experimental model of Mhy-infection by combining transtracheal and aerosol infection, using a mixture of seven local field strains.

The precise mechanisms of vaccine-associated protection are not yet fully understood, although both mucosal antibodies and cell-mediated immunity may play a role [9]. After colonizing the respiratory tract, Mhy stimulates pulmonary macrophages to secrete cytokines that induce a persistent immune and inflammatory response [5,10,25]. Pro-inflammatory cytokines, produced as part of experimental infections, activate inflammatory cells in airways and may regulate the response of numerous cell types, especially neutrophils, macrophages, and lymphocytes [10,11,16]. Cytokines, such as IL-1 and IL-4, which increased significantly in Mhy-infected lungs in relation with vaccinated animals, have been reported to activate B cell growth and differentiation in vitro [10,13]. The activation of the humoral immunity by these mediators in non-vaccinated animals would suggest a relative less role of the Th2 response in vaccinated animals, in which these cytokines significantly showed lower intensity. In addition, the decrease in the expression of these cytokines and Mhy proteins in vaccinated animals, which have been incriminated in the lymphoid activation and exertion of nonspecific mitogenic activity [13,14], would have prevented the characteristic peribronchiolar lymphoid hyperplasia of the BALT in vaccinated pigs.

Numerous studies have demonstrated the increase of IL-1, IL-8, and TNF-α in the bronchoalveolar exudate of infected animals [10,11,12,13,14,15,17], which constitute potent pro-inflammatory mediators that activate recruitment of inflammatory cells by inducing expression of adhesion molecules and stimulating the chemotaxis and activation of phagocytic cells. On the other hand, the expression of IL-10 might inhibit macrophage function and could contribute to the persistence of Mhy in the airways. The lower levels of these four cytokines in vaccinated pigs in the current study, in relation with the non-vaccinated ones, would have prevented lung damage and would have improved mycoplasma clearance from the respiratory tract. Mhy has been reported to have a suppressive influence on neutrophil function, in spite the elevated IL8 levels, which would potentially attract neutrophils in the airway spaces [10,11]. In the early stages of Mhy infection, mononuclear cells and, in a lesser extent, neutrophils, release pro-inflammatory cytokines such as IL-1and TNF-α [10,25]. In later stages [24], IL-8 upregulation was positively correlated with histological lesion score. The high IL-8 levels in Mhy-infected pigs, mainly immunolabeled in BALT areas, alveolar septa and airway exudate [14], was in accordance with the prominent infiltration of neutrophils in the airways exudate of non-vaccinated animals. Although these leukocytes have a protective role by means of phagocytosis of bacteria, the release of cytoplasmic enzymes from their lysosomes, and the increase production of inflammatory cytokines [12], may be associated with pulmonary lesions and with the perpetuation of the inflammatory stimulus. The decrease of IL-8 production in vaccinated pigs would have prevented neutrophils influx in the airways and, therefore, tissue damage.

INF-γ is a significant activator of phagocytic and bactericidal activities of macrophages in injury sites [15,17,24], along with stimulation of antigen presentation to naive T cells [26], T lymphocyte differentiation in Th1 cells [27], which is known to be an efficient mechanism to generate immunity against Mhy. The increase INF-γ expression in vaccinated pigs and its relationship with decreased mycoplasma detection and histological lung lesions reinforces these facts.

BALT hyperplasia is a characteristic histological finding during Mhy infection [1,4,5]. Mhy has a mitogenic effect on the BALT, resulting in buildup of lympho-histiocytic cells in affected lung tissue. Pro-inflammatory cytokines, such as IL-1α, TNF-α, and IL-6 have also been linked to lymphoid hyperplasia in the initial stages of the infection [10,25]. The activation of macrophages and the secretion of anti-inflammatory cytokines in the later stages, which have important immunopathological effects and influence in the progression of the disease, require further investigation.

Macrophages are critical in the production of various pro-inflammatory cytokines. These cytokines are vital in the resolution of the infection but are similarly responsible for tissue damage [5]. IL-12 acts in conjunction with IL-2 to activate T cells and induce its differentiation toward cytotoxic T lymphocytes [13,15]. The overexpression of both cytokines in vaccinated pigs can be related with the development of a Th1 response, promoting host defense against Mhy infection. In addition, IL-12 modulates several pathways in cell-mediated immunity, including the production of IFN-γ, also overexpressed in the vaccinated group, which also may be related with activation of cytotoxic T lymphocytes and up-regulation of a Th1 environment.

IL-10 is a down-regulating cytokine, mainly produced by Th2 cells and macrophages, that inhibits antigen-presenting cells, suppresses the secretion of IL-1, TNF-α, IL-12 by mononuclear cells, and exhibits inhibitory effects on the production of IFN-γ by Th1 cells [1,3,13,16]. These findings are in agreement with the significant higher numbers of IL-10-labelled cells in non-vaccinated pigs, in relation with IL-12 and IFN-γ-labelled cells. The low concentration of these latter two cytokines during Mhy infection has been hypothesized to lessen viral clearance in pigs coinfected with swine influenza virus and porcine reproductive and respiratory syndrome virus [17,26], which has been related with the potentiation and persistence of others bacterial and viral pathogens in the porcine respiratory tract during PEN [11]. Altogether, the interaction of Mhy with antigen-presenting cells seems to produce a significant reduction of IL-12 and IFN-γ by restricting cell-mediated immune response. In this context, the high expression of IL-12 and IFN-γ in lung tissue of vaccinated pigs, suggests that vaccination promoted Th1 cell differentiation, which was associated with a decrease of both the load of Mhy detected in the lung, and the intensity and extension of lesions.

Apart from the fact that Mhy adhesion to host cells is of paramount significance in pathogenesis, the exact mechanisms underlying the immunopathogenesis and cytokine induction during PEN are still unclear. Alveolar macrophages phagocytose and neutralize foreign organisms that reach lower airways, and generate oxygen radicals, cytokines, lysozyme, complement, and other products. The production of IL-12 by alveolar macrophages influences neighboring dendritic cells activation and Th1/Th2 differentiation [28,29]. IL-2 and IL-12 appear to be a requirement for producing optimal Th1 responses and to play a critical role in supporting cell-mediated immunity [13,29]. Finally, IL-12 induces the production of IFN-γ by T and NK lymphocytes and, since IFN-γ increases the production of IL-12 by macrophages, would suggest positive feedback between both cytokines in perpetuating a Th1-based response [13,17]. IL-10 expression is linked with inhibition of Th1 lymphocytes and macrophage function, which is essential for the protection against Mhy infection, suggesting that this is hypothetically one mechanism of eluding or delaying the host’s immune response [11,30]. Since IL-2, IL-12, and IFN-γ, which expression followed a similar histological pattern in vaccinated animals of this study, are potent up-regulators of immune responses [5,23], the activation of Th1 and macrophage cytolytic activity, would seem to have played an important role in Mhy-clearance from the respiratory tract of vaccinated pigs.

IL-5 and IL-3 are two relevant cytokines produced by Th2 lymphocytes which have been associated with epithelial hypertrophy and metaplasia of mucin-producing cells, stimulation of mucin MU-C5AC secretion, cellular infiltration and hyperplasia of the BALT during Mhy infection [31]. In addition, mycoplasma-lipoproteins have been demonstrated to induce IL-17A which, in conjunction with IL-8, promotes the neutrophilic infiltration in the lung parenchyma of infected pigs, enhancing tissue damage [32,33,34]. Although in the current trial the expression of these cytokines increased in the Mhy-infected pigs, in relation with uninfected controls, vaccination did not trigger their overexpression.

Thacker et al. [9] demonstrated the presence of less TNF-α in vaccinated animals after experimental infection with Mhy. As the production of this cytokine is induced in macrophages of the BALT after contact with Mhy and, since its overexpression has been associated with the induction of inflammation and tissue damage, this is in concordance with our results and previous studies [9,35] in which vaccination reduced the infiltration of macrophages, the secretion of TNF-α, and the amount of Mhy cells in the respiratory tract of pigs after challenge.

## 5. Conclusions

In conclusion, the results of this study suggest that Mhy induces a mixed Th1/Th2 immunity in non-vaccinated animals, and promotes Th1-response, by means the overexpression of IL-2, IL-12, and IFN-γ in vaccinated animals, which was related with protection against the infection. These outcomes, combined with the decrease of macroscopic and histological lesions observed in vaccinated animals, suggest the importance to improve Th1 response in the immunization strategies aimed at controlling Mhy infection.

## Figures and Tables

**Table 1:** Gross, histological, immunohistological and clinical findings in controls and Mhy-infected pigs.

Group	Pig n°	Gross Lesions	Histological Lesions		Mhy-Antigen	Clinical Signs
			BALT	Alveolar-MØ		
C0	1	0	0	0	0	0
	2	0	0	0	0	0
	3	0	0	1	0	0
	4	0	0	0	0	0
	5	0	0	0	0	0
	6	0	0	1	0	0
	7	0	0	0	0	0
	8	0	0	0	0	0
C1	9	0	0	0	0	2
	10	0	0	0	0	1
	11	0	0	1	0	0
	12	0	1	1	1	0
	13	0	1	1	0	0
	14	0	0	0	0	11
	15	0	0	1	0	0
	16	0	0	0	0	0
C2	17	23	4	2	2	4
	18	24	4	3	2	11
	19	14	4	2	2	12
	20	4	2	2	2	2
	21	37	4	3	2	1
	22	11	3	3	2	16
	23	28	4	2	2	3
	24	12	2	2	2	1

C0: sterile Friis modified liquid broth; C1: vaccinated/infected; C2: un-vaccinated/infected. Gross lesions evaluated according with the percentage of lung affected. BALT hyperplasia score evaluated according with Livingston et al. [4]; 0: absent; 1: one or more lymphoid nodules; 2: lymphoid nodules extended through the muscularis mucosa; 3: perivascular and peribronchiolar lymphoid hyperplasia; and 4: perivascular and peribronchiolar lymphoid hyperplasia invading extensive areas of the lung. Alveolar macrophages (MØ) (cells/mm^2^); 0: <20; 1: 20–40; 2: 40–60; and 3: >60. Mhy-antigen reaction; 0: negative; 1: scant; and 2: intense. Clinical signs: numbers represent the summation of daily clinical events observed.

**Table 2:** *Mycoplasma hyopneumoniae* molecular detection and quantification results. nd: not determined.

Group	Pig n°	PCR Result	Ct	Relative Copy Number/µL
C0	1	-	nd	nd
	2	-	nd	nd
	3	-	nd	nd
	4	-	nd	nd
	5	-	nd	nd
	6	-	nd	nd
	7	-	nd	nd
	8	-	nd	nd
C1	9	-	nd	nd
	10	-	nd	nd
	11	-	nd	nd
	12	+	31.8	2.90 × 10^1^
	13	+	29.7	1.20 × 10^2^
	14	+	30.1	9.18 × 10^1^
	15	+	29	1.94 × 10^2^
	16	-	nd	nd
C2	17	+	20.8	5.03 × 10^4^
	18	+	19.7	1.06 × 10^5^
	19	+	18.6	2.23 × 10^5^
	20	+	23.4	8.62 × 10^3^
	21	+	20.2	7.55 × 10^4^
	22	+	22.1	2.08 × 10^4^
	23	+	18.1	3.14 × 10^5^
	24	+	23.6	7.53 × 10^3^

## Data Availability

Not applicable.
